# Selective impairment of decision making under ambiguity in alexithymia

**DOI:** 10.1186/s12888-017-1537-2

**Published:** 2017-11-28

**Authors:** Lei Zhang, Xue Wang, Yu Zhu, Hongchen Li, Chunyan Zhu, Fengqiong Yu, Kai Wang

**Affiliations:** 1 Department of Medical Psychology, Chaohu Clinical Medical College, Anhui Medical University, Hefei, China; 2 Department of Neurology, the First Affiliated Hospital of Anhui Medical University, Hefei, China; 3 Collaborative Innovation Center of Neuropsychiatric Disorder and Mental Health, Hefei, Anhui Province China; 4 Anhui Province Key Laboratory of Cognition and Neuropsychiatric Disorders, Anhui Medical University, Hefei, China

**Keywords:** Alexithymia, Decision making, TAS-20, Ambiguity, Risk

## Abstract

**Background:**

Alexithymia is characterised by difficulties identifying and describing emotions. Few studies have investigated how alexithymia influences decision-making under different conditions (ambiguity and risk). This study aimed to examine whether alexithymia contributes to impairment in decision-making.

**Method:**

This study included 42 participants with high scores in the Chinese version of Toronto Alexithymia Scale (alexithymia group), and 44 matched subjects with low scores (control group). Decision-making was measured using the Iowa Gambling Task (IGT) and the Game of Dice Task (GDT).

**Results:**

The main findings of this study revealed selective deficits in IGT performance for the alexithymia group, while GDT performance was unimpaired when compared with the control group. In IGT, total netscores were lower for the alexithymia group compared to the control group, particularly with regard to block 5. Moreover, the alexithymia individuals selected significantly more adverse cards than the controls, indicating significant decision-making impairments.

**Conclusion:**

Alexithymia selectively influences decision-making under ambiguity.

## Background

Several recent studies have supported the hypothesis that emotions may modulate human behaviour and cognition, and many researchers have extensively examined the influence of negative emotions on higher cognitive functions [1]. From an evolutionary perspective, negative emotions, such as anxiety, serve to signal a potential threat in the environment [2]. However, inappropriate activation of the negative emotion system may contribute to the onset of some psychiatric disorders [3]. Alexithymia (ALEX) is typically characterised by difficulties describing, understanding or identifying self-other emotions. This condition was originally thought to be a syndrome exhibited by patients with psychosomatic and psychiatric disorders [4]. Currently, alexithymia is understood as a personality trait, the severity of which varies across different populations [5]. Several studies have found that individuals with alexithymia demonstrate poor performance on executive and cognitive tasks associated with prefrontal cortex [6], and are related with a decline representational function regarding decision-making [7].

Decision-making is an important and complex cognitive function including assessing and evaluating the short- and long-term costs and benefits that may be attributed to different options. In the field of neuroscience, two types of decision-making differ mainly with regard to the level of unsureness concerned as well as how much useful information is offered to the operator in terms of the consequences of decisions and their associated probabilities [8]. In some cases, consequences and possibilities are uncertain. In order to determine the viability of the options available, decision-makers must assess relevant information according to feedback based on the previous selection. The form of decision-making is described ‘decision-making under ambiguity’ and is commonly evaluated using the Iowa Gambling Task (IGT) [9]. In the IGT, choosing cards from advantageous decks results in maximum profit. Participants need to overcome an initial attraction to high-payoff decks with subsequent big punishments. Contrary to the ambiguous decision-making, for some aspects of decision, explicit information is provided concerning the possible results of various options and their associated probabilities. This is known as decision-making under risk, and is evaluated using the Game of Dice Task (GDT) [10]. In GDT, participants are asked to choose among four different choices which clearly related to winning or losing. In addition, the probabilities associated with winning are clear and stabilized before participants begin testing. Some options are related to high latency wining / losing, while other options are associated with lower potential wining / losing. In planning to win as much money as possible, participants must consider which options will lead to more benefits. Personality and emotion are important factors in risky decision making [11]. Alexithymia is a personality trait, typically characterized by inability to understand, describe and explain one’s emotions. In addition, there is evidence suggesting that the reduced ability to make emotional appraisals in alexithymia affects the performance of risk decision tasks [12, 13]. Therefore, we speculated that alexithymia could modulate risky decision making.

Neuroimaging and neuropsychological studies have identified several brain areas that are correlated with decision-making, including the ventromedial prefrontal cortex (vmPFC), the dorsolateral prefrontal cortex (dlPFC), the orbitofrontal prefrontal cortex (OFC) and the anterior cingulate cortex (ACC) [14–16]. Deficient IGT performance, for example, the preference to select adverse decks, was presented in patients with vmPFC / OFC lesions [16, 17]. Patients with vmPFC lesions exhibit a significantly greater reflection effect, choosing more gambles under the condition of losing and fewer gambles under the condition of wining [17]. Depressive patients with suicide history that were related with dysfunction of left lateral orbitofrontal cortex showed alterations in the processing of risk under conditions of uncertainty [16]. However, some articles have shown that the dlPFC has an important influence in performance on the GDT. Patients with dlPFC dysfunction showed impairment of performance on the GDT [18, 19]. Moreover, risky decision-making evaluated by the GDT, rests on activation of the dlPFC [20]. While these brain areas are associated with decision-making, they also have a significant impact on alexithymia. Several neuroimaging researches have suggested that alexithymia is associated with decreased activation of the vmPFC [21], reduced orbitofrontal cortical thickness [22] and altered cognition-related brain activity within the dlPFC [23]. Thus, alexithymia may influence the two different patterns of decision making.

Few studies have investigated decision-making under different conditions (ambiguity and risk) in alexithymia. As decision-making involves the cognitive capacity to process emotions [24, 25], this current study used the IGT and the GDT to examine whether alexithymia contributes to deficits in decision-making. We hypothesized that alexithymia would have deficits in their decision-making function. Moreover, we compared the different performances between the two decision making tasks in alexithymia. As GDT performance may be determined by some certain executive functions, such as monitoring and classification, and use of feedback without persistent tendency (as measured by the Wisconsin Card Sorting Test (WCST)) [26, 27], and in order to balance the differences in executive functions related to performance of GDT, subjects completed WCST [28].

## Methods

### Participants

Five hundred forty students who were in Grade Two and from Anhui Medical University were assessed for alexithymia using the Chinese version of the Toronto Alexithymia Scale (TAS-20) [29]. Finally, 513 valid questionnaires were returned. The sample included 284 male and 229 female with an average age of 19. The average score of the TAS-20 of 513 students was approximately 49.2. We selected the cut-off for high alexithymia – ALEX group – >60 reflecting the top quartile score; and cut-off for low alexithymia – Control group – <40 reflecting the bottom quartile score, in order to obtain a sample with as large a variance in alexithymia as possible [30]. All participants satisfied the following exclusion criteria: (a) no history of substance abuse (including alcohol), (b) no demonstrable brain disorder (e.g., epilepsy, schizophrenia, brain injury or head trauma), (c) no depressive or anxiety disorder (i.e., exclusion of participants who scored ≥41 as measured using the Self-Rating Depression Scale [SDS]; exclusion of participants who scored ≥40 as measured using the Self-Rating Anxiety Scale [SAS]).

Finally, the selected sample consisted of 42 students scored higher than 60 in the alexithymia group (mean = 64.31; SD = 2.63) and 44 students scored lower than 40 in the control group (mean = 36.82; SD = 2.34). Participants ranged from 20 to 21 years of age. In these two groups, the ratio of female to male was similar. (Table 1). This study was approved by the ethics committee at Anhui Medical University. Written informed consents were obtained from all the participants and ¥20 was accepted for their participation.

**Table 1:** Demographic data of alexithymia and control groups

	Alexithymics(*n* = 42)	Controls(*n* = 44)	t/χ2	*P*
Age (years)	19.23(0.48)	19.25(0.44)	0.12	0.91
Gender (male/female)	21 m / 21f	23 m / 21f	0.04	0.83
Education (years)	14.19(0.40)	14.25(0.44)	0.66	0.51
SDS	34.23(5.77)	32.07(6.29)	1.22	0.23
SAS	33.18(4.74)	30.78(5.57)	1.36	0.17
TAS-20 (score)	64.31(2.63)	36.82(2.24)* **	52.3	0.000
WCST				
wrong responses	19.79(8.81)	18.23(6.37)	0.93	0.35
perseverative response	38.38(7.37)	37.86(6.21)	0.35	0.73
perseverative errors	12.78(5.32)	12.69(6.42)	0.07	0.95

*p < .05, **p < .01and ***p < .001

### Procedure

#### SDS and SAS

SDS is a self-reported 20-item questionnaire that generally considered a reliable instrument for measuring depressive symptoms in the general population [31]. Each item is this questionaire is scored from 1 (rarely) to 4 (frequently), according to the frequency that symptoms affect the subjects’ life. SAS is a 20-item measure developed to assess the frequency of anxiety symptoms [32]. The four-point scoring method was applied in this questionnaire (from 1 point to 4 points), according to severity of symptoms. A higher score indicates more serious anxiety symptoms.

### Decision-making under ambiguity

The computerised version of the Iowa Gambling Task is a usual method of measuring decision making under condition of uncertainty [33]. The IGT assesses the ability to choose between outcomes that yield high incomes with the high risk of future loss, low incomes with small risk of loss. In this task, subjects were asked to accumulate as much money as possible by selecting one card from each of the four decks (A, B, C and D) at a time, until 100 cards were chosen. Following each card selection, the message “You get 100” or “You get 100 but lose 200” appeared on the screen immediately. Decks A and B were disadvantageous decks. These decks yielded high instant returns and higher losses, leading to long-term negative consequences. In contrast, decks C and D were beneficial. These decks yielded small instant gains, but the losses are small, resulting in long-term positive consequences. In addition, there were additional differences between the four decks. Although both A and B decks were unfavorable decks, the choices of the deck A were penalized in 50 % of the trials, whereas deck B choices were penalized in 10 % of the trials. Similar differences were evident with regard to decks C (50% losses) and D (10% losses). The instant losses for deck D were larger than those for deck C [34]. The netscore was measured by calculating the scores associated with card selections from advantageous decks (C + D) and disadvantageous decks (A + B). One hundred choices were equally divided into 5 blocks. Calculation of netscore for each block was assessed changes in decision making over time. Typically, the netscore, (C + D) – (A + B), is used to analyse the results obtained from the IGT.

### Decision-making under risk

In the computerised GDT, a virtual shaker and a single die appears on the screen [10]. Participants were asked to maximise their fictitious start-up funds (€1000) within 18 dice throws by selecting one of four different options. Before each throw, participants had to choose either a single die or one die from two, three or four dice combinations. Each choice is related to particular fictitious gains and losses, based on the probability of occurrence: €1000 gain/loss for the choice of a single number with probability of winning 1:6; €500 gain/loss for a combination of double numbers with probability of winning 2:6; €200 gain/loss for a combination of triple numbers with probability of winning 3:6; and €100 gain/loss for a combination of quadruple numbers with probability of winning 4:6. The winning probability attributable to the different selections may be deduced easily by referring to the ratio of occurrence (1:6, 2:6, 3:6, and 4:6). For instance, if a participant bets on a single number “one”, and one is thrown, the participant wins €200; however, if a two, three, four, five, or six is thrown, the participant loses €200. The first two choices were classified as risky decisions for lower winning probabilities; the two latter choices were classified as safe decisions for higher winning probabilities. Furthermore, after each option, some changes appeared on the screen including the number of remaining dice, gain or loss and capital change. To analyse risky decisions, the netscore was calculated (the number of safe options minus the number of risky options) to analyse task performance. In addition, the frequency of each option was analysed separately.

The order across the two tests was counterbalanced by a Latin-square design and all subjects could rest if they felt fatigued. At the end of the experiment, subjects were asked whether they were interested in the experiment and what kind of experiences they felt during the experiment. The whole experiment lasted approximately 20 min.

### Statistical analysis

Statistical analysis was carried out with SPSS version 17. A repeated measures analysis of variance (ANOVA) was used, with the trial blocks (IGT) and different types of choices (GDT) as the within-subjects factor and study group as the between-subjects factor, to examine the influence of performance on decision-making tasks under different conditions. Post hoc test was used to test the effects of the different process of decision-making in the IGT. A one-way ANOVA was performed to analyse the effects of four different types of choices in GDT. The statistical significance level was *p* < .05.

## Results

No statistically significant differences were found in terms of age, gender, educational level, SDS or SAS score between the two groups. Notably, there was no significant difference in WCST scores between the two groups. The results illustrated that the ALEX group have a similar executive function when compared to the control group (Table 1).

### IGT and GDT

**Fig. 1:**
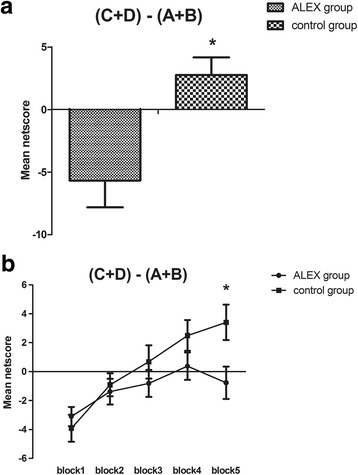
Total netscore during the Iowa Gambling Task (IGT) and netscore of the five blocks in the task between two groups. (C+D)-(A+B): the number of advantageous choices minus the number of disadvantageous choices *p < .05, **p < .01and ***p < .001

To examine the IGT and GDT performances, a repeated measures ANOVA was conducted, with group as between-subjects factor and task as the within-subjects factor. There was a significant main effect for task (*F*_1,84_ = 23.87, *p* = .000), and for a group by task interaction (*F*_1,84_ = 8.68, *p* = .004). According to the post hoc LSD tests, the netscores of IGT in the two groups were significantly lower compared to the netscores of GDT (*p* = .001). Pair wise comparisons indicated a remarkable difference between the total IGT netscores of the two groups (*t*_1,84_ = −2.13, *p* = .036) (Fig. 1a). The result showed that the beneficial decks selected in the control group were more than those in the ALEX group. Contrary, the difference was not statistically significant in the netscores of the GDT between the two groups (*t*_1,84_ = 1.49, *p* = .14). Furthermore, in the ALEX group, the significant difference the was found between IGT and GDT netscores (*t*_1,41_ = 4.99, *p* = .000). However, in the control group, the difference was not statistically significant (*t*_1,43_ = 1.54, *p* = .13).

### Decision-making in the IGT

#### Netscore in the IGT

In order to investigate the IGT performances in detail, a repeated measure ANOVA was conducted, with group as the between-subjects factor and block as the within-subjects factor. The main effect for group was significant (*F*_1,84_ = 4.02, *p* = .048), showing that performances in the control group outperformed those in the ALEX group; for block (*F*_4,336_ = 9.48, *p* = .000), indicating a dynamic process of change in IGT; and for a block by group interaction (*F*_4,336_ = 5.21, *p* = .025). In the IGT experiment, the change of decision strategy is reflected by the change curve. Post hoc LSD tests revealed that the difference was not statistically significant between adjacent blocks on the netscore of the ALEX group (*p* = .19, *p* = .66, *p* = .36, *p* = .38). However, there was a significant effect of decision process between block 1 and block 2 and between block 3 and block 4 on the netscore of the control group (*p* = .03, *p* = .046), showing that the decision making strategies of the control group changed significantly with the process of the task. In addition, simple effect analysis of netscores of five blocks suggested statistical difference for block 5 between the two groups (*p* = .014) (Fig. 1b).

### Individual deck level preference

**Fig. 2:**
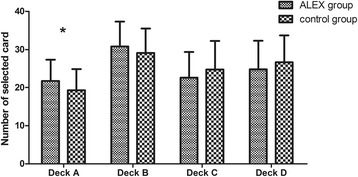
Number of four types of cards selected for two groups during the Iowa Gambling Task (IGT). *p < .05, **p < .01and ***p < .001

In order to examine the trend of individual deck level in IGT, a repeated measure ANOVA was used. There was a significant main effect for deck (*F*_3,252_ = 5.91, *p* = .02), and for a group by deck interaction (*F*_3,252_ = 3.17, *p* = .048). Simple effect analysis of performances on the four types of decks between the two groups indicated that significant statistical difference existed in the score of deck A (*p* = .044). The results suggested that the Alex group chose more adverse options than the control group (Fig. 2).

### Decision making in the GDT

#### GDT netscores

**Fig. 3:**
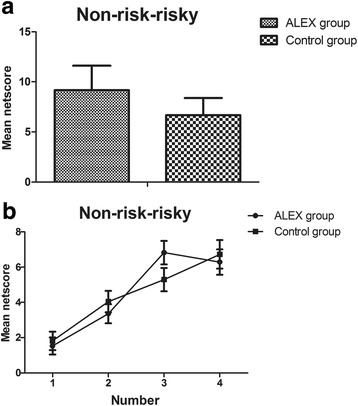
Netscore and mean frequency of each alternative during the Game of Dice Task (GDT) between two groups. Non-risky-risky: the number of safe choices minus the number of risky choices

By comparison,no significant difference was found in the netscores of the GDT between the two groups (Fig. 3a). The collecting dates were analysed by repeated measurement analysis of variance, with group as the between-subjects factor and option as the within-subjects factor. For option, main effect of option was significant (*F*_3,252_ = 18.73, *p* = .000). However, the main effect of group had no significant statistical difference (*F*_1,84_ = .95, *p* = .33), and no significant option by group interaction (*F*_3,252_ = .97, *p* = .42). With regard to the rate of each type of selection, none of the simple comparisons had significant differences between both groups: one single number (*t*_1,84_ = −0.42, *p* = .68); two numbers together (*t*_1,84_ = −0.84, *p* = .40); three numbers together (*t*_1,84_ = 1.65, *p* = .10); four numbers together (*t*_1,84_ = −0.41, *p* = .69) (Fig. 3b).

Following the selection of a risky option, this study explored the utilisation of negative feedback (loss) in GDT to choose a safe option. Those subjects who chose a risky choice and accepted negative feedback once or more were selected. Therefore, a total data of 74 subjects were collected and analysed. Both groups had no significant difference concerning the utilisation of negative feedback (*t*_1,72_ = 1.67, *p* = .10). No significant correlation existed between the netscore of GDT and the utilisation of negative feedback (ALEX group: *r* = 0.02, *p* = .87; control group: *r* = 0.17, *p* = .085). The utilisation of positive feedback (gains) was in those who decided on a safe choice in choosing a safe choice again in the following trial. Those subjects who selected a safe choice and accepted positive feedback once or more were selected. Therefore, a total data of 82 subjects were collected and analysed. The two groups differed significantly in the aspect of the utilisation of positive feedback (*t*_1,80_ = 1.27, *p* = .21). In addition, no significant correlation existed between the netscore of GDT and the utilisation of positive feedback (ALEX group: *r* = 0.12, *p* = .11; control group: *r* = 0.20, *p* = .067).

## Discussion

This current study investigated two decision-making situations using participants with alexithymia and a control group. The main findings show that participants with alexithymia demonstrated selective deficits in IGT performance but unimpaired GDT performance when compared with the control group. Total IGT netscores were lower for the ALEX group than for the control group, particularly with regard to block 5. Furthermore, both two groups showed distinct trends of individual deck level for the IGT: the ALEX group chose more adverse options than the control group, indicating significant decision-making impairments.

The ALEX group performed worse than the control group in the IGT. The results suggest that participants in the ALEX group were preferred instant income and were difficult to develop an effective strategy in the long run. This result corresponds with previous findings that suggested a correlation between alexithymia and deficits in decision-making abilities, indicating that alexithymia may be a critical personality trait underlying decision-making deficits [7]. Poletti et al. found that total IGT scores did not differ between alexithymic and non-alexithymic patients with Parkinson’s disease. However, significant differences emerged across the third block of the IGT [35]. Alexithymic gamblers chose less advantageously in this task than non-alexithymic gamblers in the IGT. The more severity of alexithymia appeared, the more severity of the deficit in decision-making abilities damaged [7]. As anxiety and depression are recognised as factors that may impair decision-making abilities [36, 37], subjects who had high levels of depression and anxiety were excluded through the SDS and the SAS. Comparable to the findings of previous research [38, 39], alexithymia appears to be a stable personality trait rather than a phenomenon [7]. In addition, it may be noted that total IGT scores were lower for the ALEX group than for the control group, particularly with regard to block 5. This finding is supported by the consolidation-attenuation model, in view of a mounting evidence that suggests a correlation between alexithymia and deficits of information processing concerning emotional arousal [40], as well as emotional-based memory [41]. Thus, during the process of experiment, alexithymia individuals may have declining ability of continuous learning and difficulty to use experience to guide future behaviour which would otherwise lead to attenuated learning.

Importantly, for the IGT, the findings of this current study show that the ALEX group selected more deck A cards than the control group, indicating a significant decision-making impairment. The clinical manual of IGT states that decks A and B are adverse options, and that selections of deck A is avoided by most “neurologically intact” individuals [34]. As previously outlined, selections of deck B may not discriminate effectively between the decision-making performances of subjects in both two groups, while continuous selections from deck A are more indicative of pathological risk-taking [34]. Thus, while deck B was sensitive to risky decision-making, deck A was more sensitive to decision-making impairment. Current research suggests that there may be evident correlations between personality characteristics and deck A selections among non-clinical and clinical populations, for example, individuals with trait anxiety, substance abuse/dependence and obsessive-compulsive disorder [18, 42, 43]. Our result is in accordance with the contention that alexithymia related differently to in the selection of deck A compared to controls.

In contrast to the results from the IGT, no performance differences in the GDT were found between the alexithymia group and the control group. Previous studies that examined patients with various diseases as well as normal participants showed a correlation between disadvantageous decisions in risky decision-making and low executive performance, including task switching, flexibility of cognition and categorisation as measured by the WCST [44]. A patient who underwent brain resection for glial cyst was examined using the cognitive tasks, executive function tests and intelligence test. Results showed selective deficits in decision making and executive functions, compare with other cognitive components [45]. Furthermore, additional studies found that participants with intact executive functions showed no impairment in performance in the GDT [46, 47]. Consequently, previous research suggests that the association is most attributable to participants’ utilisation of information concerning the possible options of GDT that obviously involved planning, monitoring and modification of favorable strategies. In summary, we hypothesised that executive functions may influence performance in the GDT. In support of this hypothesis, there were no statistically significant differences between the two groups in WCST, and no differences in performance in the GDT. This current study outlined that performance of the GDT was also related to dealing with feedback with regard to winning and losing. The adverse selections of patients with eating disorders were obviously more than those of the control group, and safer decisions of patients were often less in response to positive feedback [48]. Similar results were shown in patients with attention deficit hyperactivity disorder (ADHD) [27]. Therefore, performance deficits in the GDT were hypothesised to be related to disadvantageous utilisation of feedback. In this current study, no significant difference was found in dealing with feedback or in other performance measures for the GDT between two groups.

Although alexithymia individuals may have impairment of feedback processing in the IGT, the ability of using feedback implicated in the GDT remained intact. Research has shown that this dissociation can been explained in that the two decision making tests depend on the two processes to a certain extent [49, 50]. Several studies have indicated that the difference between GDT and IGT is similar to a shift from explicit to implicit knowledge [47, 51]. In addition, while executive functions are not as important as the use of feedback for exploring some rules in the IGT, they are particularly important for the use of favorable strategies in the GDT [52]. Thus, future studies will need to investigate the dissociation between decision-making under ambiguity and risk using the methods such as electrophysiology and neuroimaging.

There are some limitations of this study. First, as the findings of several studies involving both general and clinical populations have suggested, alexithymia is a stable personality trait [53, 54]. While studies of alexithymia involving general populations may offer more information reflecting alexithymic trait, patient studies can focus specifically on the association between alexithymia and susceptibility to psychiatric symptoms. Second, our final sample included participants who ranged from 20 to 21 years old. It is well documented that age has an influence on personality, although the development of personality is relatively mature and stable after 18 years of age. Further investigations are required to determine the role of alexithymia among different age groups in general population and in psychosomatic and psychiatric disorders.

## Conclusion

Our current study shows that alexithymia selectively influences decision-making under ambiguity. Our findings support the hypothesis that emotions may modulate human behaviour and cognition, especially decision making. In addition, we sought to reveal how emotions affect decision making under different conditions and other cognitive functions. Importantly, the findings extend the view that personality traits have impact on decision making. Further studies may utilise clinical populations to clarify the results and neural mechanisms through the method of functional magnetic resonance imaging and event-related potentials.

## Abbreviations

ACC: Anterior cingulate cortex
ADHD: Attention deficit hyperactivity disorder
dlPFC: Dorsolateral prefrontal cortex
GDT: Game of dice task
IGT: Iowa Gambling task
OFC: Orbitofrontal prefrontal cortex
SAS: Self-rating anxiety scale
SCRs: Skin conductance responses
SDS: Self-rating depression scale
SMH: Somatic-marker hypothesis
TAS-20: Toronto alexithymia scale
vmPFC: Ventromedial prefrontal cortex
WCST: Wisconsin card sorting test
